# Lipid-based nanoparticles as a promising treatment for the skin cancer

**DOI:** 10.1016/j.heliyon.2024.e29898

**Published:** 2024-04-19

**Authors:** Parisa Golestani

**Affiliations:** Department of Biology, Mashhad Branch, Islamic Azad University, Mashhad, Iran

**Keywords:** Lipid-base nanoparticles, Nanoliposomes, Skin cancer, Drug delivery

## Abstract

The prevalence of skin disorders, especially cancer, is increasing worldwide. Several factors are involved in causing skin cancer, but ultraviolet (UV) light, including sunlight and tanning beds, are considered the leading cause. Different methods such as chemotherapy, radiotherapy, cryotherapy, and photodynamic therapy are mostly used for the skin cancer treatment. However, drug resistance and toxicity against cancer cells are related to these treatments. Lipid-nanoparticles have attracted significant interest as delivery systems due to non-invasive and targeted delivery based on the type of active drug. However, the stratum corneum, the outer layer of the skin, is inherently impervious to drugs. Due to their ability to penetrate the deep layers of the skin, skin delivery systems are capable of delivering drugs to target cells in a protected manner. The aim of this review was to examine the properties and applications of nanoliposomes used in the treatment and prevention of numerous types of skin cancer.

## 1 Introduction

The skin is the largest organ in the human body, which protects the external surface of the body effectively. The most important task of skin is physical, chemical, and safety protection between the body, and the external environment [1]. Any anomaly happening in this layer will result in diverse sorts of skin insults, and cancers are one of the most important. Radiation therapy, chemotherapy, and surgery have made significant advances in treating cancer over the past few years. There has been limited success with conventional therapeutic strategies for the treatment of cancer due to systemic adverse effects, drug resistance, and suboptimal drug concentrations at the site of the tumor [2]. Conversely, topical application of drugs or active pharmaceutical ingredients (API) is restricted by the physical and chemical barriers of the skin. For effective treatment of skin diseases, APIs should be formulated in a well-coordinated vehicle capable of regulating epidermal penetration, inhibiting irritation, and providing maximum protection against skin diseases [3]. The potential role of nanoparticles in the treatment of various types of cancer has attracted increased attention in recent decades [[4], [5], [6], [7], [8]]. Nanoparticles can target drug delivery system, meaning they can be designed to encapsulate chemotherapy drugs or targeted therapies and release them selectively in cancer cells. This ensures that a higher concentration of the drug is delivered to the tumor site while minimizing the side effects on surrounding healthy tissue. Additionally, nanoparticles can also act as contrast agents in imaging modalities, aiding in early detection and diagnosis [[9], [10], [11], [12], [13], [14]].

Skin disorders and skin cancers can be treated with different phospholipid vesicles since they concentrate drugs at the site of action and can minimize adverse effects by predicting the drug's systemic absorption [15,16]. There are many types of nanoparticles, but lipid-based nanoparticles are unique biocompatible carriers that have attracted much attention in the treatment of cancer. Because of their unique characteristics, these nanoparticles have several applications in biomedicine, including the encapsulation and transport of hydrophilic as well as hydrophobic drugs, and the controlled and targeted release of drugs [[17], [18], [19], [20]]. In addition to improving transdermal delivery, lipid-based nanoparticles can be utilized as models for skin membranes [21].

Liposomes can transport drugs well through the skin. However, they have several problems like susceptible phospholipids to oxidation that leads to the breakdown and collapse of its biolayer and leakage of encapsulated drugs [22]. Therefore, new generations of lipid-based nanoparticles were created, including transferosomes, phytosomes, ethosomes, niosomes, etc, and have been widely considered for transdermal delivery. This review investigates the characteristics of various types of lipid-based nanoparticles and their administrations in skin cancer drug delivery.

### 1.1 Skin cancer

Globally, skin cancer ranks fifth among cancer types, and its burden is continually increasing [23]. Skin cancer is a common dangerous tumor in humans [24]. Melanoma (cancers arising from the dysfunction of melanocytes) and non-cancer skin cancers (originating from epidermal cells) are the two most common types of skin cancer [25]. About 95 % of skin cancers are non-melanoma skin cancers (NMSCs) caused by genetic and natural factors. Non-melanoma skin cancer includes a variety of distinct cancerous types, but they can be divided into two most important subtypes. Basal cell carcinoma (BCC) and cutaneous squamous cell carcinoma (SCC) account for 99 % of all NMSCs [26].

BCCs, the most common type of skin cancer, account for 80 % of all keratinocyte carcinomas that are diagnosed in clinics [27,28]. The primary site of BCC is the back of the hands and the face of middle-aged or elderly patients. It is thought to be caused by follicular stem cells within the epidermis. As with most skin cancers, BCC is influenced by ultraviolet radiation (UV) [28,29].

One of the most common forms of keratinocyte cancer is cutaneous squamous cell carcinoma (CSCC) [28,30]. As a result of precancerous injuries known as actinic keratoses (AK) or sun-powered keratoses, CSCC may develop [28,31]. Actinic keratosis occurs as a widespread condition in intensely sun-exposed body ranges [28]. UV light exposure is the most significant risk factor for the development of AK and SCC. Due to their intense exposure to UV light, the head and neck regions are the most frequently affected by this disease [32,33]. A number of variables can be associated with the occurrence of CSCC, including natural variables such as daily exposure to UV light, human papillomavirus, which may be diagnosed in skin cancers such as BCC and SCC, and long-term exposure to chemicals such as bitumen or tar derivatives [24].

The third most common type of skin tumor is melanoma [34] that is caused by harm to melanocytes [35], and in comparison to different skin insults, accounts as it were for 1 % of all dangerous skin tumors and considered as a majority of skin cancer deaths (up to 80 %) [26,36].

Several methods are widely used to treat skin cancer including excision surgery, chemotherapy, radiation therapy, cryotherapy, immunotherapy, targeted therapy, and photodynamic therapy. Nonetheless, each has advantages and disadvantages, for instance chemotherapy as a main treatment suffering from poor bioavailability, insufficient solubility and permeability, low therapeutic efficacy and severe side effects and off-targeting. Today nanotechnology is frequently used for cancer therapy to reduce the disadvantages of traditional methods. Recently, many researchers are trying to overcome the toxicity barrier and provide a therapeutic method based on nanoparticles for the effective skin cancer therapy.

### 1.2 Nanotechnology in skin cancer treatment

The field of nanotechnology, which uses nanoscale materials, has significant capacity to improve the efficacy of cancer treatments as drug delivery vehicles and therapeutic agents. There are different types of nanomaterials that are frequently used for different purposes of cancer treatment. One of the applications of nanotechnology is for the delivery of chemotherapy agents that lack specific targeting to the tumor region, which in turn leads to poor uptake and accumulation of drugs in different organs, resulting in adverse effects. In addition, therapeutic agents with low water solubility and permeability, poor half-life, low bioavailability, and inadequate stability in physiological conditions cannot deliver the necessary therapeutic effects, so nanotechnology has demonstrated successful results in improving these shortcomings [37]. In the non-metastatic form of skin cancers, drug delivery to the site of tumor can potentially prevent systemic toxicity and reduce side effects. In spite of this, the insufficient permeability of therapeutic agents to enter the skin has been considered as one of the major obstacles. Hence, nanomedicine has a broad potential to improve the problems through enhanced permeation and retention (EPR) effect and engineering with other materials [38]. Skin cancer has been treated using various nanoparticles so far, including lipid-based nanoparticles, protein-based nanoparticles, polymers, superparamagnetic iron oxide nanoparticles (SPIONs), dendrimers, carbon-based nanoparticles, and inorganic nanoparticles. In recent years, lipid-based vesicles have attracted increasing attention as a method of administering drugs to the skin, but their use remains a challenge. In this review, we examine the most commonly used lipid-based nanoparticles for treating skin cancer.

### 1.3 Lipid-based drug delivery systems

In addition to improving drug effectiveness, nanotechnology can be applied to improve drug pharmacokinetics and decrease drug side effects [39]. In 1950, transdermal drug delivery was introduced. By using the transdermal system, drugs are transported from the surface of the skin into the bloodstream by passing through several layers of the skin [40]. In the past two decades, liposomes were created as a well-known lipidic -based vesicular carrier method [41]. Bingham first described lipid-based vesicles in 1965, hence the name "Bingham Bodies" [42]. It is especially important to develop venous drug delivery systems for targeted delivery of hydrophilic and hydrophobic drugs, which enhance the bioavailability of drugs, delay their metabolization rate, increase the half-life of drug circulation in the body, improve stability, and reduce the toxicity of encapsulated drugs [43].

In recent studies, it has been demonstrated that drug delivery into the skin is closely related to the size of lipid vesicles. It is therefore unlikely that vesicles with a diameter of 600 nm or greater can transport the encapsulated drug to deeper layers of the skin, but may remain on the stratum corneum and may form a lipid layer upon drying [44,45]. The transport of drugs to deeper layers of the skin is possible with nanovesicles with a diameter of 300 nm or less. The largest drug deposition occurs in each layer of the skin and epidermis when nanovesicles have a diameter of 70 nm or less [44,46]. The transepidermal lipid pathways are generally able to absorb nanoparticles with diameters less than 6–7 nm, while aqueous pores can absorb nanoparticles with diameters less than 36 nm. The transfollicular pathway can be significantly penetrated by nanoparticles in the size range of 10–210 nm [15,47].

Several mechanisms and applications have been reported because of their ability to enhance the delivery of percutaneous drugs into deeper layers of skin. There are three possible routes for a permeant to pass across the epidermis when applied to the skin. Transcellular routes involve lipids associated with proteins inside corneocytes, intercellular routes involve lipids associated with proteins inside corneocytes, and appendageal routes involve hair follicles and sebaceous glands. There may be a variety of mechanisms of drug transport depending on the nature of the drug [48,49].

It is believed that transdermal enhancement of hydrophilic drugs occurs as a result of various mechanisms, including (i) enhancing drug thermodynamic activity - the encapsulated drug vesicles are adsorbable and fused to the skin surface. Afterwards, a thermodynamic activity gradient is created, thereby increasing the diffusion pressure for drug penetration, which is a driving force for drug penetration across stratum corneum; (ii) modification of the electrical charge on the surface of ionic drugs; (iii) solubilization of sebum by vesicles to facilitate follicular delivery; and (iv) pore pathways for large water-soluble molecules loaded into lipid nanoparticles. It is possible to enhance the transdermal delivery of hydrophobic drugs by disrupting the lipid bilayer of the stratum corneum (SC) first. By disrupting the densely packed lipid bilayer, structural modification of the stratum corneum enhances permeation by filling up extracellular spaces. In the second step, nano-sizing enhances transdermal permeation. Third, changing drug partition into skin layers; and fourth, hydrating skin and dilation of the SC intercellular channels—niosomes alter the barrier properties of stratum corneum, which enhances skin hydration by reducing *trans*-epidermal water loss. This loosens the tightly packed structure, lyses the membrane with lysozyme, and releases the drug [48,50,51]. Furthermore, lipophilic permeants can be delivered via a follicular pathway. As an endocytosis enhancer, nonionic surfactants play a crucial role in penetrating into intercellular lipids [48].

### 1.4 Lipid-base nanovesicles used for local treatment of skin cancer

**Fig. 1:**
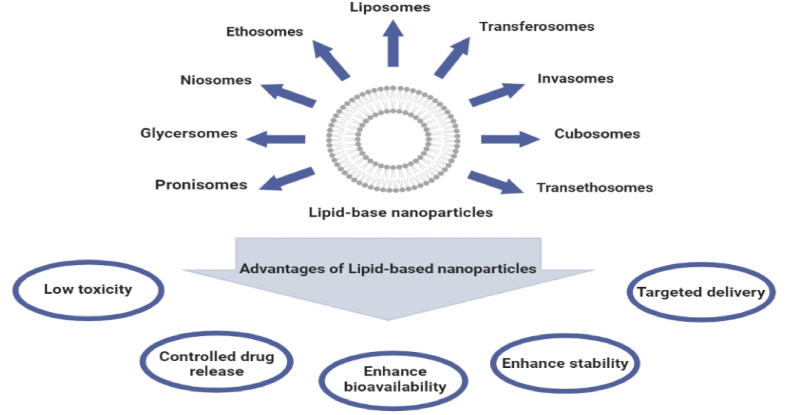
Different types of lipid-based nanoparticles and advantages of transdermal delivery with lipid-based nanoparticles.

There are many therapeutic applications for lipid-based nanoparticles at the nanoscale. As a result of their important and unique properties, they are ideal for medical use, as they have a much higher surface-to-mass ratio than other colloidal particles, as well as the ability to bind or absorb and transport other compounds [52]. Lipid nanoparticles are unique systems for topical and transdermal drug delivery [47]. In the following, we will examine their structure and application of these particles in the local therapy of skin tumors. The major lipid-based nanoparticles and their importance in transdermal delivery were shown in Fig. 1. Moreover, Table 1 lists various forms of lipid-based nanovesicles using in skin disorders therapy.

**Table 1:** Different type of lipid-base nanoparticles for local administration and treatment of skin cancer.

Nanocarrier type	Encapsulated Compound	used as a model	Aim	Ref.
**Liposome**	aloe-emodin (AE)	Human Nonmelanoma Skin Cancer Cells	Improving drug penetration into the skin, accelerating the cell death of A431 and SCC25 cells in a short period of time	[146]
	siRNA	UACC-903 human melanoma cell line	Significant reduction of BRAF protein expression level	[147]
**Transfersome**	Rose Bengal (RB)	SK-MEL28 melanoma cells	RB-TF protected RB against adverse photodegradation during 24 h of direct light exposure, while increasing the amount of RB in the epidermis, thereby reducing the growth of melanoma cells.	[148]
	5-fluorouracil(5-FU)	HaCaT (Nonmelanoma) cancer cell lines	5-FU-loaded transfersomes showed the highest cytotoxicity on the HaCaT cell line and also improved the vesiculation of 5-FU for local delivery.	[149]
	Honokiol (HK)	B16F10 melanoma	This formulation has negatively charged surface, high encapsulation efficiency, controlled release profile, downregulation of TGF-β signaling, diminished expression signal — CD47, and decreased expression of CD133 marker.	[150]
	paclitaxel (PTX)	B10F16 melanoma	This formula increased the deformability and the penetration of PTX-CT into the skin, thence decreasing the growth of the tumor.	[151]
**Ethosome**	curcumin (CUR)	A375 human melanoma cell lines	This formula transported the medicate molecules to the more profound layers of the skin and caused the passing of A375 cells by the mechanism of apoptosis.	[152]
	Mitoxantrone (MTO)	A375 cells	this formulation had high in vitro permeability, remarkable cytotoxicity, and a high anti-cancer effect.	[153]
	Silver nanoparticles (AgNPs)	A431 skin carcinoma cells	This formula entered the deep skin and released free radicals that kill cancer cell by depolarization of the mitochondrial membrane.	[154]

### 1.5 Liposomes

Liposomes are phospholipid bilayer vesicles (50–100 nm) with similar structure to the biological membranes [53]. Liposomes are primarily composed of lipids and phospholipids [54]. Liposomes comprise a lipid bilayer composed primarily of natural and synthetic phospholipids. Other components including cholesterol or saturated lipids, can provide a favorable performance for the liposomal formulation. There are a few liposomes that contain additional components like antioxidants, carbohydrates, proteins, or sterols [54]. During the formation of a bilayer, the hydrophilic heads of the liposome would align with the water compartment, while the lipophilic tails would align with the center of the vesicle [55]. According to their size and number of layers, they can be classified as multilamellar, oligolamellar, or unilamellar. It is possible to encapsulate hydrophobic or amphiphilic compounds in lipid bilayers, whereas hydrophobic compounds can be encapsulated in an aqueous core, thereby reducing their toxicity [56].

Liposomes are biodegradable, biocompatible, nontoxic, non-immunogenic [57], and flexible [58] materials and they are prepared using natural materials during a simple process [59]. Also, PEGylated or stealth liposomes have been developed to reduce clearance and increase the half-life of blood circulation [58]. A liposome's bilayer structure allows it to act as an enhancer of drug penetration in target tissues owing to its exceptional compatibility with the skin surface [60]. They can apply extraordinary features after topical application, such as improved drug deposition within the site of action. Despite the favorable advantages, liposomes have disadvantages such as a decrease in encapsulation efficiency with increasing diameter, physical instability during drug encapsulation drug leakage, short half-life, low solubility, oxidation, and hydrolysis in some cases can be unavoidable [58,59].

The majority of cell membranes are made up of phospholipids, so liposomes are being investigated as a means of systemic and topical administration and are being proposed as a cosmetic or therapeutic product. In addition, liposomes successfully cover wounds and after application create a moist environment on the wound surface, which is very beneficial for wound healing [61].

Mandeep Marwah and et al., formulated Naringenin in liposomes in the presence of Tween 20, then suspended it in hydroxyethyl cellulose (HEC) and hydroxypropyl methylcellulose (HPMC) gels [62]. In another study, they formulated epigallocatechin gallate (EGCG) in liposomes with expanded stacking of Tween 20 [63]. In both studies, Tween 20 in liposome layers decreased the diameter of liposomes, increased their flexibility and reduced drug encapsulation. In this case, drug encapsulation may have been reduced due to Tween 20 competing for space within the bilayer or increased drug dissolution capacity. Liposomes reduced drug release, were absorbed into epidermal keratinocytes and skin fibroblasts within 2 h, and improved the treatment of cancer [62,63]. Subcutaneous or topically applied liposomes loaded with soybean lunasin and amaranth unsaponifiable matter (UM + LunLip) were effective in treating melanoma tumor-developing groups since they moderated cell proliferation and induced apoptosis [64]. It was observed by Anup Jose et al., that curcumin-containing liposomes penetrated up to 160 nm into the skin and inhibited tumor growth in a B16F10 animal model after iontophoretic co-delivery of anti-STAT3 siRNA and curcumin using cationic liposomes [65]. As well, Korani and et al., improved the efficacy of Bortezomib by encapsulation of them in liposomal structures [66]. They could improve the anti-cancer property of Bortezomib in the treatment of melanoma. Also, liposomes can be modified with different agents for improving the function within the body. For example, liposomal doxorubicin functionalized with TAT peptide improved antitumor efficacy in B16F0 tumor models compare to nan-functionalized liposomes [67]. Along with the unique advantages of liposomes, there are problems in using them to deliver drugs to the skin which mainly related to entrapment efficacy, penetration, fluidity and stability. Liposomes are not able to penetrate the stratum corneum and accumulated on upper layer of skin. Therefore, in order to improve their skin penetration, there were changes in their structure and composition, which led to the emergence of new types of lipid-based nanovesicles, which are widely used in drug delivery today.

**Fig. 2:**
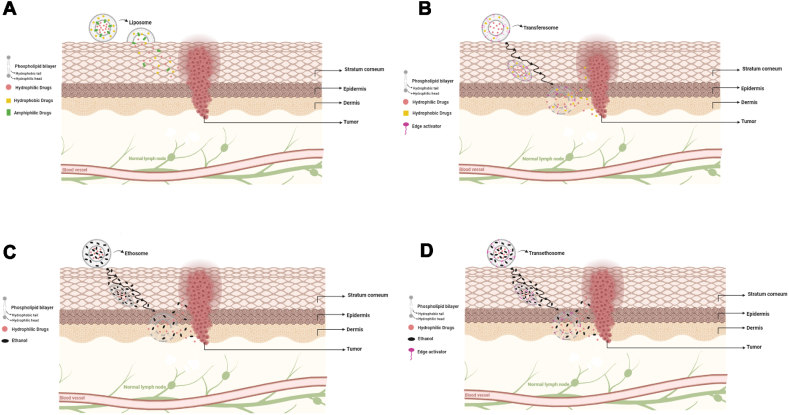
The scematic representation of skin peneteration mechanism of varipus lipid based nanoparticles. (A) Liposome, (B) transferosome, (C) ethosome, and (D) transethosome.

According to general consensus, conventional liposomes are unable to penetrate the skin layers and remain on the surface of the skin acting as a depot formulation [68]. Through the "collision complex transfer process" observed in other biological systems, the liposomes may directly interact with the skin and exchange the drug, or they may release the free drug which can penetrate through the skin. Lastly, liposomes have the ability to induce structural changes in the stratum corneum, which facilitates the absorption of drugs (see Fig. 2A) [69].

### 1.6 Transferosomes

The transferosome is a novel lipid-based vesicule that is an excellent carrier of drugs in order to control their distribution in the body. Sodium cholate, phospholipids, and an edge activator are the components of transferosomes [70]. Various therapeutic agents such as protein, insulin peptides, proteins, hormones, corticosteroids, and anticancer drug can be transferred with transferosomes [71].

Transferosomes are biocompatible, biodegradable and highly deformable nanoparticles which can quickly penetration through the subcutaneous tissue. The peneteratin of transferosomes occure at a rate largely higher than other lipid-base nanoparticles [72,73]. When transfersomes are placed on the surface of intact skin, can go across a skin and intercellular space of the epidermal cells, under the transepidermal osmotic gradient, they can penetrate deeply into the skin and also protect their encapsulated drug from rapid clearance, which in turn causes increasing circulation and bioavailability [74].

They are capable of acting both as carriers of drugs and as penetration enhancers, and in addition stimulate skin hydration [75,76]. Like liposomes, transferosomes can trap both hydrophobic and hydrophilic drugs in phospholipid bilayer and aqueous space [70]. In addition, they can encapsulate high or low molecular weight drugs [77]. One of main drawbacks of these vesicles include their chemical instability due to their susceptibility to oxidative damage, and expensive formulations. Alternatively, hydrophobic drugs are difficult to load into transferosomes without compromising their deformability and elasticity [43,78].

Recently, R-carvedilol-loaded transfersomes were prepare. These transferosomes caused the low levels of drug rlease and high penetration of the drug in the layers of the skin. In addition, topical treatment with R-carvedilol-loaded transfersomes could reduced severe skin inflammation caused by ultraviolet rays (UV) [79]. 5-fluorouracil (5-Fu) largly used for the treatment of actinic keratosis and non-melanoma skin cancer but 5-FU showed low transdermal penetration, so its anticancer effect was reduced after topical application. In 2015, a research group et al., using Span-80 and Tween-80 as edge activators, provided a distinct definition of transferosomes for the encapsulation of 5-Fu. Vesiculization of 5-FU as a transferosomal gel 5 -Fu enhanced its topical delivery became non-irritating and allowed 5-Fu to penetrate the skin, thus providing a suitable treatment for skin cancer [80]. Moreover, previously reported that lipidic vesiculas containing edge activator in their structures were able to effectively penetrated to basal epidermis of the skin. Evaluating of these structures containing siRNA (lipoplexes) on melanoma cells indicated that apoptosis was increased as a result of the inhibition of B-RAF expression in murine sarcoma viral oncogene homolog B1 (BRAF) cells. These results indicated that an edge-activated lipidic structures could effectively carry nucleic acids through the stratum corneum and dermis [81].

The extent of flexibility and partition coefficient are the two main parameters that determine the movement of transfersomes across the cutaneous surface (see Fig. 2B). Hydration force governs the passage of transferosomes through the stratum corneum layer. As a result of the lower fluid content of the stratum corneum, a fluid gradient is created. Cevc et al. explained the passage through the skin in terms of osmotic strength. Through the stratum corneum, the active ingredient is easily released and diffuses through different layers of the skin until it reaches the bloodstream. By deforming themselves, transferosomes pass through the intercellular space of the stratum corneum. Transferosomes have not been reported to be capable of permeating the skin without altering its shape. Upon interacting with the disturbed layer of the stratum corneum, the vesicular carrier transethosome makes its way through the stratum corneum into the deeper layers of the skin by adhering to lipid lamella. Due to ethanol and edge activators present in vesicles, vesicles are capable of passing through narrow intercellular pathways easily due to their elastic nature [40].

### 1.7 Ethosomes

Ethosomes are modified lipid nanovesicles and composed of ethanol, phospholipids, and water that frequently used for delivery of treapeutic agents to different layers of the skin. Ethanol as a permeation enhancer constitute a relatively high part of autosomes (20–45 %). The unique properties of autosomes, such as elasticity and deformability, are mainly due to the incorporation of a high percentage of ethanol. Consequently, they penetrate deeper into the skin and increase drug penetration and absorption. Overall, ethanol increases the fluidity of the epidermis, decreasing the thickness of the lipid layers, and increasing the flexibility of the vesicular structure. As a result, ethanol allows the drug to penetrate further into the epidermis. On the other hand, ethanol gives a negative charge to autosomes, reduces the estimation of vesicles and finally increases the bioavailability of the drugs [82].

Various studies have shown that the autosomal system has a high capability as an effective nanocarrier for the delivery of API to the skin [83]. The key advantage of ethosomes is their capacity to carry drugs into the deep skin layer [84]. Song et al., described a new generation of autosomes that aim to combine the advantages of deformable liposomes and autosomes [84,85]. Therefore, to improve the permeability of ethosomes, a new generation of binary ethosome and transethosome was created [84,86]. In several studies, it was found that ethosomal systems loaded with drugs can maintain their size and stability for up to one year during the storage period [87].

A previous report indicated that the efficacy of autosomes loaded with metformin has a higher skin penetration rate than free metformin gels. It seems that high concentration of ethanol has better results for skin penetration and produces significant antitumor properties on skin cancer [88]. Despite its promising potential for treating skin cancer, sonidegib (SNG) has low bioavailability and adverse side effects. Amr Gama et al., developed ethosome gel of SNG to enhance its biological effects to treatment of cancer skin cancer. SNG-containing autosomes with a higher penetration in the deep skin layer showed significant bioavailability and thus antitumor activity. Hence, athosome can provide favorable therapeutic benefits of SNG for skin cancer treatment [89]. In addition to chemical compounds, natural compounds are also encapsulated in autosomal structures. Recently indicated that athosomal form of Brucine (BRU), which the use of this natural product has demonstrated significant anticancer activity against a variety of skin disorders, including melanoma [90]. Moreover, ethosomes containing curcumin showed significant deposition on the skin and had maximum release within 12 h. This allows the curcumin to stay in the deep skin layer for a long time, which helps kill melanoma cells without causing pain on the skin [91]. It is evident from these studies that drug-loaded ethosomes are a promising therapeutic strategy for the transdermal delivery of drugs.

As ethanol is present in ethosomal preparation, it intercalates on lipids present in the stratum corneum, increasing membrane permeability. It is the flexibility of ethosomes that allows them to successfully deliver drugs inside of the cells after they have fused with the membrane (see Fig. 2C) [40].

### 1.8 Transethosomes

Transethosomes are a new generation of non-invasive lipid vesicles that derived from derive from transfersome and ethosome composition and more were designed with the aim of increasing the ability of vesicle penetration [92,93]. In order to possess the advantages of both transfersomes and ethosomes, transethosomes should have the same composition as ethosomes and be capable of enhancing penetration and activating the edge of the membrane. Like other lipid vesicles, transethosomes are biodegradable and biocompatible and do not contain any toxic pharmaceutical additives and release their compounds very slowly and gradually [84]. Ethanol and propylene glycol/oleic acid are the main compounds for the penetration of transautosomes. Ethanol acts on corneodesmosomes and causes the release of corneocytes, thus making it easier to penetrate the skin layers, while penetration enhancers (surfactants or various agents) help to remove the barrier of the stratum corneum and loosen the dense protein structure and prepare the skin for more permeability makes drugs and vesicles more porous [[94], [95], [96], [97]]. Probably, the synergy between ethanol and surfactant present in Transethosomes causes small size, excellent flexibility, and better permeability to the skin [84,98] and this vesicle can effectively pass through narrow intercellular pathways [40] and drug releases into the blood circulation, indicating a systemic effects. Transethosomes are atractive systems for transdermal drug delivery [99]. While transautosomes have many advantages, they also have some disadvantages, which include misfortune of items during transfer from alcohol and water environments, skin irritation and allergic reactions associated with contact dermatitis, and an ineffective arrangement of vesicles that may connect transautosomes [96,97]. So far, several transethosomes have been developed and used for transdermal delivery of bioactive compounds. Ahmed A. H. Abdellatif et al., indicated that delivery of celecoxib (CXB) using transethosomes have lower cytotoxic effect on normal skin cells and significantly increased drug concentration in the skin, so that by inhibiting cyclooxygenase-2 (COX-2) resulted in the reducing cell growth and induction of apoptosis in cancer cells [100]. Furtheremore, transethosomes containing Dacarbazine (DAC) and tretinoin were introduced which were able to delivery drugs to the treatment of cutaneous melanoma. These dual drug-loaded transethosomes demonstrated the success of transautosomes for dual delivery of transdermal drugs which in turn has great potential for the development of synergistic effects of multi drug delivery systems in the near future [101].

With the help of vesicle components, transethosomes enhance the permeability of the components of the free drug through the skin (see Fig. 2D). Transethosomes are penetrated by ethanol since edge activators alone are not sufficient to penetrate the lower layers of the skin. Transethosomes contain ethanol that disrupts the phospholipids in the stratum corneum, resulting in fluidization. An increase in fluidization results in an increase in intracellular space, which in turn increases penetration. As a result of transethosomes, the pores are hydrated and enlarged. Due to the smaller diameter of the pores, the edge activator is able to utilize its deforming properties to help squeeze the molecule through the stratum corneum. The dermis is reached after passing through the corneum and the feasible epidermis [102].

### 1.9 Niosomes

Niosomes are non-ionic lipid base particles that composed of non-ionic surfactants and cholesterol [103]. Non-ionic surfactants, uncharged single-chain surfactant are the essential parts of niosomes, and due to the surface charge of vesicles is low, and vesicles collected and precipitate easily [103,104] As well, cholesterol as one of main components of niosomes affecting membrane fluidity. However, the concentration of cholesterol in niosomes is lower than liposomes, which lead to higher encapsulation efficiency in niosomes [104]. The stability and properties of niosomes depend on different factores including type of surfactant, composition of phospholipids bilayer, storage temperature, enteraped drug preoperty, type of detergents, and method of their production [105].

There are several types of nonionic surfactants available, all of which are important in the preparation of niosomes; they need to be biodegradable, biocompatible, non-irritating, and non-immunogenic [103,106]. They can protect active ingredients from degradation, maintain sustained release, improve drug penetration into the skin, and reduce skin irritation and enhanced the duration of drugs [107]. In addition to these positve features of niosomes, aggregation, fusion and leaking considered as a main problems of them [108]. However, niosomes are promising drug delivery platform and potentially have been applied for delivery of pharmacological agents against various diseases.

Niosomes have been studied for transdermal, oral, parenteral and topical routes to improved therapeutic effectiveness. Amino-artemisinin derivative such as Artemisone possess antitumor activity. Niosomal form of Artemisone indicated significant cytotoxicity against melanoma cells [109]. In a preclinical model of B16F10 melanoma, Gude et al. encapsulated cisplatin in niosomal structures and evaluated its antimetastatic ability. As a result of their research, they found that niosomal cisplatin exhibited effective anti-metastatic properties and was less toxic than free cisplatin [110].

Sherif Ashraf Fahmy et al., designed and optimized a niosomal vesicular nano stage loaded with Ozonated olive oil (OL) (OL/NSs) that caused accomplished a sustained release behavior for OL and improved skin penetration and applied the increased anticancer action when assayed on A375 cells [111]. Donatella Paolino et al., proposed an innovative niosomal system made up of α,ω-hexadecyl-bis-(1-aza-18-crown-6) (Bola), Span 80 and cholesterol for topical delivery of 5-FU that was named Bola-niosomes. Bola-niosomes increased the percutaneous penetration of 5-FU about 8- and 4-folds into the skin and diminishment of cellular practicality of cancer cell lines utilized [112].

**Fig. 3:**
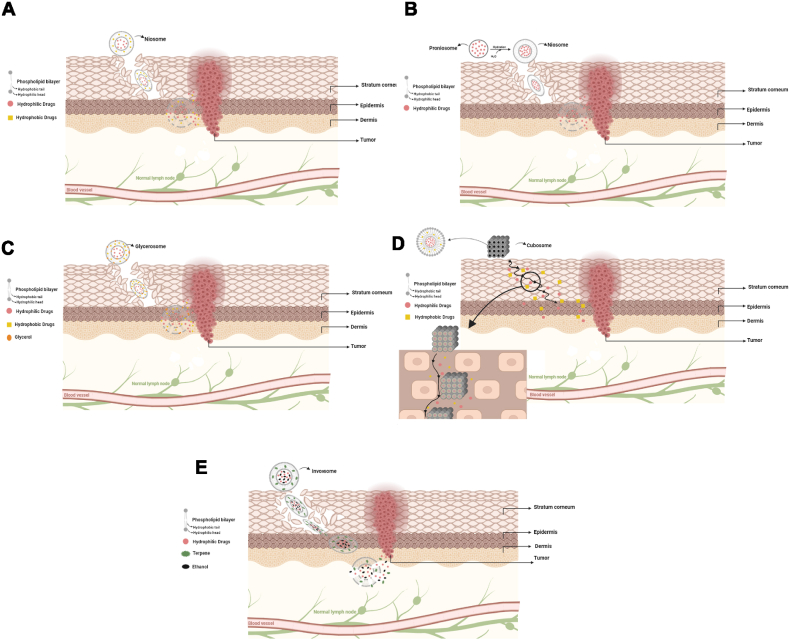
The scematic representation of skin peneteration mechanism of varipus lipid based nanoparticles. (A) Niosome, (B) pronoisome, (C) glycerosome, (D) cubosome, and (E) invaosome.

Numerous mechanisms have been proposed in literature for the interaction between niosomes and skin, including diffusion through skin, interlinking with skin lipids, and altering stratum corneum structure, which increases skin permeation (see Fig. 3A). A surfactant acts as a permeation enhancer and directs the fusion between vesicles and stratum corneum in the niosome structure [113]. Due to their surfactant properties, niosomes are capable of altering the structure of the SC, causing the layer to become looser and more permeable [114].

### 1.10 Proniosome

Proniosomes are dry surfactants coated carrier formulations with water solubility [115]. Generally, proniosomes are non-hydrated niosomes formed after hydration [116]. Dry proniosomes possess a higher degree of stability than niosome. Proniosome minimizes the instability problems of niosome that caused by aggregation, fusion and leakage. Proniosomes have considerable physical stability during the storage and improved transportation, distribution, and storage properties [117,118]. There are many different types of non-ionic surfactants that can be found in proniosomes, including lecithin, alcohol (ethanol, methanol, isopropyl alcohol) and chloroform [119]. Providing a suitable vehicle for drug delivery, proniosomes prevent hydrolysis or oxidation of encapsulated drugs, increase penetration rates into target tissues, and reduce toxicity [115,120]. As with other lipid counterparts they can both lipophilic and hydrophilic drugs through topical and transdermal [121]. Jampala Rajkumar et al. formulated 5-FU in proniosomal gel for the topical treatment of melanoma. Gels loaded with 5-FU showed high cytotoxicity in A-375 human melanoma cells and reduced tumor size after [122].

As demonstrated in Fig. 3B, an advantage of proniosomes is their ability to attach to the stratum corneum, convert to niosomes after hydration, and permeate into the skin through the stratum corneum. This results in a higher level of skin penetration [123].

### 1.11 Glycerosome

Glycerosomes are another form of bilayer nano-vesicles which mainly made of phospholipids, and various amounts of glycerol (10–30 % v/v). However, they may contain other components such as glycerophospholipids, phosphatidylcholine, sphingomyelins, cholesterol, surfactants gelatin, chitosan, cyclodextrin, hyaluronic acid. Glycerol is main constituent of these lipids serve as penetration enhancer and improves stability, fluidity, entrapment efficacy of nanoliposome bilayer then liposomes and thus increases skin penetration [124]. Glycerosomes have been administrated via oral, intranasal, topical, ophthalmic, and pulmonary routes and have been used with high efficiency and remarkable outcomes in the treatment of melanoma, wounds, and as well used in cosmetics and skincare products [[125], [126], [127], [128], [129], [130]]. However, vesicles with less than 20 % glycerol have reduced viscosity, permeability, and flexibility. Increasing the amount of glycerol in the vesicles delays drug release because glycerol disturbs the osmotic balance between the receptor and the donor [125,128]. As a result, the viscosity of glycerosome leads to increased stability, but on the other hand, it may delay the time for the vesicles to reach the skin surface [125,131]. Transdermal study of glycerosomes were evaluated by Manca et al., for diclofenac sodium delivery. They showed that permeation efficiency of diclofenac improved with glycerosomes than the conventional liposomes [132]. The effects of oxidative stress on the skin were also evaluated using glycerosomes containing quercetin. It was found that glycerosomal quercetin had a strong ability to reduce free radicals and to protect human keratinocytes from hydrogen peroxide damage [133]. In addition, Shadab Md et al. developed a glycerosome gel containing plumbagin, which caused the drug to accumulate in the upper dermis and increased antioxidant activity. Thus, glycerosomes may be a promising carrier for treating skin disorders [129].

Glycerol was found to promote drug accumulation and permeation into phospholipid vesicles because it acts as a moisturizing agent, which disrupts the orderly structure of the skin (see Fig. 3C) [126]. Due to lipid disruption and reorientation in the skin, this change resulted in increased fluidity of SC lipids. In glycerosomes, glycerol acts as a modifying agent by modifying the polar groups of the lipids, increasing their fluidity and facilitating the diffusion of nanovesicles into the skin [134].

### 1.12 Cubosomes

Cubosomes are nano-lipid particles that composed of biocompatible amphiphilic lipids and used as carriers in controlled release of hydrophilic, lipophilic and amphiphilic drugs similar to liposomes. Other component of cubosomes are [135], poloxamer 407, glycerol, glyceryl monooleate (GMO), carbopol 934P, polyvinyl alcohol (PVA), stabilizing operator (polyvinyl alcohol), and water. In aqueous solution under high energy dispersion such as shearing or high-pressure homogenization or sonication bicontinuous cubic liquid crystal structures are formed [136,137]. Cubosomes possesses great properties such as high surface area and cubic crystal structure, high stacking capacity, high drug payloads, thermodynamic stability, the ability to functionalization for controlled released, are considered as promising nanocarrier for diverse drug-administration routes. Due to the presence of non-ionic surfactants, these nanoparticles have higher physicochemical stability than liposomes and do not undergo hydrolysis in aqueous solutions. Cubosomes can be administrated by several route like oral, percutaneous, and intravenous. Also, cubosomes are used in topical applications and cancer treatment for delivery of drugs with low bioavailability and remarkably enhances skin permeation [136,137]. Resveratrol is a well-known therapeutic polyphenol with effective anticancer properties against melanoma. However, low bioavailability of resveratrol strongly limited its the therapeutic activities. Therefore, Kurangi et al., designed resveratrol-loaded cubosomes to ameliorate its transdermal its activity. Formulated resveratrol in cubosomes increased skin penetration and deposition of drug without irritating effects [138]. Moreover, the performance of cubosomes as cutaneous delivery systems evaluated by delivery of anti-inflammatory drugs (NSAIDs) that frequently used in treatment of percutaneous inflammation. But, NSAIDs are irritating if taken for an extended period. Reported that used of cubosomes for delivery of NSAID's enhanced its anti-inflammatory activity and reduced possible side effects [136].

As demonstrated in Fig. 3D, cubosomes have a structural management similar to that of skin, allowing them to be compressed through the pores of the stratum corneum so that they can penetrate deeper into the skin [139].

### 1.13 Invasome

Invasomes (Fig. 3E) are another lipid-based vesicular structure have the same basic constituents as liposomes, but they also contain terpene in their structure. Terpenes, phospholipids, and ethanol are the most units of the invasomes structure. The presence of terpenes and ethanol in the structure of invasomes act as permeation enhancers and increase permeability, and deformability and enhancing its permeability into deeper layers of skin. They are non-toxic vesicles that can easily incorporate drugs (both lipophilic and hydrophilic) and interestingly increase transdermal the permeability of incorporated agent [140]. The main disadvantages of invasomes are high production cost, and drug leakage, and also hydrolysis or oxidation of phospholipid may occur, which affects the stability of enosomes [41]. To dated, several attempts have been made in the field of drug delivery to the skin with these structures, which have had satisfactory results. Heba F. Salem et al., formulated vismodegib (VSD), an anti-skin cancer agent, in invasomes to improve the its bioavailability and dermal penetration. Invasomes containing VSD significantly increased the permeability, bioavailability, and efficacy of VSD. Invasomes loaded with VSD can serve as an alternative to oral administration for the treatment of skin tumor because these structures significantly enhanced VSD transdermal flux into deep layers of the skin [141]. Also, Prashant Bhardwaj et al., created β-caryophyllene assembled invasomes of 5-FU that significantly increased the antioxidant property and drug deposition of 5-FU in the skin [142]. Table 2 compares the property of lipid-based nanoparticles in comparison to liposomes.

**Table 2:** The property of lipid-based nanoparticles in comparison to liposomes.

Types of nanoliposome	Size (nm)	Properties compare to liposome	Figure	Ref.
Liposome	50–200	Lower bioavailability than other nanoliposomes, Low ability to deliver drugs to the skin		[59,155]
Transferosome	>300	Higher flexibility, Higher transdermal permeability.		[[156], [157], [158]]
Ethosome	150–200	Higher transdermal permeability, negative zeta potential, higher absorption rate		[84,159]
Transethosome	150–350	Higher entrapment efficiency, flexibility, and transdermal permeability		[160]
Niosome	200–350	Easer storage and lower leakage		[103,104]
Glycerosome	80–100	More fluidity, Higher transdermal permeability		[125]
Cubosome	100–500	Higher capacity for encapsulation of hydrophilic, lipophilic, and amphiphilic molecules.		[161]
proniosome		More efficient, higher chemical stability		[115,162]
Invasome	11–13	Higher transdermal permeability		[163,163]

The literature survey indicates that a variety of types of skin have been used for permeation studies, including human (female) abdominal skin obtained after plastic surgery, albino (male) Wistar rat skin, porcine skin, and rabbit skin after fatty tissue is removed. It is possible for the invasomes to combine with the lipids of the skin and alter the distribution of SC, thereby loosening the tight lipid junctions of the skin [[143], [144], [145]].

## 2 Conclusion

There is a growing interest in the use of nanovesicles for the delivery of targeted drugs today. An investigation was conducted on the use of lipid-based nanoparticles to deliver drugs transdermally to different types of skin cancer in this article. Lipid-based nanovesicles offer several advantages compared to conventional treatments. They allow for targeted drug delivery, improving bioavailability and minimizing side effects. Additionally, their small size allows them to penetrate deeper into tissues and target specific cells, leading to enhanced therapeutic outcomes. By using drug carriers, new drug delivery strives to maintain a constant and effective level of the drug in the body, reduce adverse effects as well as localize the action of the drug. Local treatment of skin cancer with the help of these nanoliposomes can be a good alternative to other skin cancer treatment methods. None of the drug delivery systems alone can provide all the necessary criteria for drug delivery, but efforts are underway through new approaches.

## CRediT authorship contribution statement

**Parisa Golestani:** Conceptualization, Investigation, Writing – original draft, Writing – review & editing.

## Declaration of competing interest

The authors declare that they have no known competing financial interests or personal relationships that could have appeared to influence the work reported in this paper.
